# The Association between Excess Body Mass and Disturbances in Somatic Mineral Levels

**DOI:** 10.3390/ijms21197306

**Published:** 2020-10-03

**Authors:** Weronika Banach, Karolina Nitschke, Natalia Krajewska, Wojciech Mongiałło, Oskar Matuszak, Józef Muszyński, Damian Skrypnik

**Affiliations:** 1 Faculty of Medicine, Poznań University of Medical Sciences, Fredry St. 10, 61-701 Poznań, Poland; weronika.wb98@gmail.com (W.B.); karolinanitschke@wp.pl (K.N.); krajnat@gmail.com (N.K.); wojmzl@gmail.com (W.M.); oskar.piotr.matuszak@gmail.com (O.M.); joozef.muszynski@gmail.com (J.M.); 2 Department of Treatment of Obesity, Metabolic Disorders and Clinical Dietetics, Poznań University of Medical Sciences, Szamarzewskiego St. 82/84, 60-569 Poznań, Poland

**Keywords:** obesity, excess body mass, body mass index, minerals, elements

## Abstract

Background: Obesity and excess body weight are significant epidemiological issues, not only because they are costly to treat, but also because they are among the leading causes of death worldwide. In 2016, an estimated 40% of the global population was overweight, reflecting the importance of the issue. Obesity is linked to metabolism malfunction and concomitantly with altered mineral levels in the body. In this paper, we review alterations in somatic levels of iron, calcium, magnesium, copper, iodine, chromium, selenium, and zinc in relation to excess body mass. Methodology: An electronic literature search was performed using PubMed. Our search covered original English research articles published over the past five years, culminating in 63 papers included for study. Results: The reviewed papers presented correlation between obesity and hypomagnesemia and hypozincemia. They also indicated that patients with excess body mass present increased body copper levels. Studies have similarly indicated that obesity appears to be associated with lower selenium levels in both blood and urine, which may be correlated with the decline and weakening of defenses against oxidative stress. It has been found that decreased level of chromium is connected with metabolic syndrome. Chromium supplementation influences body mass, but the effect of the supplementation depends on the chemical form of the chromium. It is hypothesized that obesity poses a risk of iodine deficiency and iodine absorption may be disrupted by increased fat intake in obese women. A range of studies have suggested that obesity is correlated with iron deficiency. On the other hand, some reports have indicated that excess body mass may coexist with iron excess. The relation between obesity and body iron level requires further investigation. Calcium signaling seems to be disturbed in obesity, due to the increased production of reactive oxygen species and low level of fast troponin isoform responsible for mediating calcium sensitivity of muscle relaxation. Correlation between excess body mass and calcium levels needs further research. Conclusions: Excess body mass is associated with alterations in mineral levels in the body, in particular hypomagnesemia and decreased selenium (Se) and zinc (Zn) levels. Chromium (Cr) deficiency is associated with metabolic syndrome. Obese patients are at risk of iodine deficiency. Excess body mass is associated with elevated levels of copper (Cu). Data on the association between obesity and iron (Fe) levels are contradictory. Obesity coexists with disturbed calcium (Ca) signaling pathways. The association between obesity and body Ca levels has not been investigated in detail.

## 1. Introduction

Obesity is an important epidemiological problem in the modern era. It was estimated in 2016 that 1.9 billion adults were overweight, as defined by having a body mass index (BMI) of at least 25 kg/m^2^. Of these, 600 million individuals were obese (BMI ≥ 30 kg/m^2^). This 1.9 billion cohort constitutes 40% of the global population [1,2]. Should this obesity epidemic continue at the same rate, approximately half the world’s adult population will be overweight or obese by 2030.

Obesity generates huge costs: it is estimated that health care costs for obese individuals are 44% higher than for individuals with normal body mass. The costs associated with obesity treatment are estimated at two billion dollars per annum [3]. When adiposity increases, so does mortality, as obesity is now the fifth leading cause of death worldwide.

Obesity is associated with alterations of mineral levels in the body [4]. Recent studies have identified iron (Fe), calcium (Ca), magnesium (Mg), zinc (Zn), copper (Cu), selenium (Se), iodine, and chromium (Cr) as minerals that are prone to excess-body-mass-associated disturbances in their somatic levels. However, studies of obesity and Fe have presented contradictory results. Trials on excess body mass and Ca levels remain inconclusive, while the number of studies on excess body mass, Se, and iodine has been quite limited to date [5,6,7,8]. Minerals—particularly Fe, Ca, and Mg, but also Zn, Cu, Se, and iodine—play an essential role in a range of metabolic processes and in the body’s energy balance. Obesity is a life-threatening disease, currently reaching epidemic proportions, which is associated with altered levels of these minerals. There is thus a great need to present the current state of knowledge concerning the associations between excess body mass and changes in somatic mineral levels [9,10].

Excess body mass is associated with significantly detrimental metabolic disorders, such as type 2 diabetes mellitus, cardiovascular diseases (CVD), cancer, and nonalcoholic fatty liver disease (NAFLD) [5,11]. Thus, full knowledge of the disturbances in somatic mineral levels associated with excess body mass seems to be crucial.

The aim of this paper was to review the current literature in order to identify disturbances in the levels of minerals associated with excess body mass.

## 2. Methodology

Papers meeting the following criteria were included: original research articles written in English and dealing with excess body mass and minerals. Articles describing research on both humans and animals were included. Nonoriginal research articles, duplicated articles, review articles, case reports, or publications in languages other than English were excluded. We sought current and contemporary data, so 87% of the included articles were published after 2015.

In order to present important data not found in papers published after 2015, a range of older studies were included (13%). We searched PubMed to identify the appropriate sources. In total, 976 matching studies were found. After reviewing the abstracts, 891 were excluded as not associated with the aim of the review. The full texts of the remaining 85 articles were analyzed by five of us, and a total of 62 papers were finally included in this narrative review.

**Figure 1:**
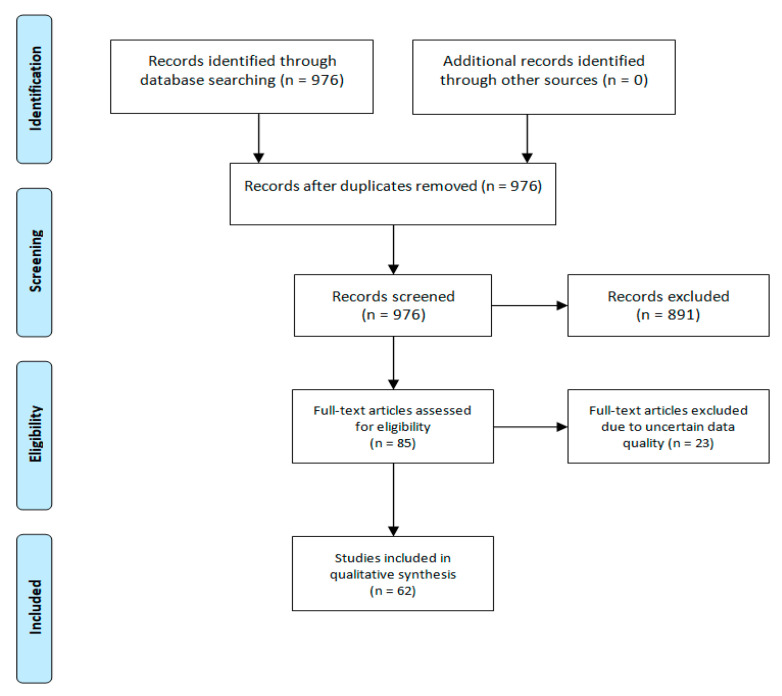
Flow diagram of the review.

We excluded 23 papers due to uncertain data quality (Figure 1). The level of evidence (LOE) has been graded on a seven-point scale (Ackley, B. J.; Swan, B. A.; Ladwig, G.; Tucker, S. Evidence-based nursing care guidelines: Medical-surgical interventions; Elsevier: St. Louis, MO, USA, 2008; p. 7. (https://libguides.winona.edu/c.php?g=11614&p=61584)).

## 3. Results and Discussion

### 3.1. Iron

Fe is an essential mineral component for all living organisms; however, its body level can be disturbed in patients with excess body mass. Fe is a key component of oxygen-carrying proteins, such as hemoglobin and myoglobin [12] (LOE V), which are essential for growth and cell differentiation [13] (LOE IV). In a longitudinal study of 1613 pregnant women from rural China supplemented with Fe, Jones et al. suggested that maternal obesity during pregnancy adversely affects maternal and neonatal Fe status, and that inflammation seems to be the underlying mechanism [13]. This high-quality cohort study presents the detrimental effect of excess body mass on body Fe level.

Over 40 years ago, a connection between obesity and Fe deficiency was described by Wenzel et al. who investigated 25 obese adolescent boys, 36 obese adolescent girls, and their matched nonobese peers. Although the study population was small, the authors concluded that the serum Fe levels of obese boys and girls were significantly lower than those of normal-weight boys and girls [11] (LOE IV). An increased prevalence of Fe deficiency, or a risk of Fe deficiency, has been presented among overweight and obese children and adolescents by Hutchinson in a review that evaluated studies of the relationship between iron and excess body mass in children and adolescents, published in December 2015. Overweight and obesity are particularly unfavorable for children and pose a serious health issue for this group [14] (LOE V). Children require increased Fe levels as they are rapidly growing and developing, and this is especially true for females [14]. It has been suggested that overweight and obese girls begin to menstruate earlier, which might contribute to the higher incidence of Fe deficiency in this group [14]. Fe deficiency impairs cognitive function, behavior, and exercise performance; obese children have less developed cognitive functions than children of normal weight, and some hypotheses have suggested that Fe deficiency associated with obesity may impair brain development in this group [5,14] (LOE V). It is believed that Fe-deficient overweight female children will become Fe-deficient adolescents and adults [14]. The data on the detrimental effect of Fe deficiency on the mental and physical development of obese adolescents present an appalling vista. The results of the studies reviewed by Hutchinson constitute a significant argument for obesity prevention and treatment programs in this age group.

In adults, Fe deficiency is potentially adverse, especially during pregnancy. Data suggest that lowered Fe availability at the placenta occurs in obese pregnant women, and similarly, excessive weight gain during pregnancy can result in a lower Fe status in newborns [13].

Three mechanisms are proposed as potential causes of obesity-related Fe deficiency: the systemic low-degree inflammation typical of obesity, the increased blood volume due to the increased weight of fat tissue, resulting in higher Fe demands, and dietary Fe deficiencies [5].

The subclinical inflammation induced by excess body mass stimulates higher levels of circulating hepcidin, which may lead to a deficiency of Fe in the blood, called hypoferremia [15] (LOE IV). Such a relationship has been presented by Cepeda-Lopez in a study of 62 healthy nonanemic women. Hepcidin is a main regulator of Fe metabolism and is primarily produced by the liver in response to inflammation and increased Fe accumulation [15]. The study of Citelli et al. on C57BL/6J mice fed either a control diet or a high-fat diet indicated that obesity induces hepcidin gene expression in adipose tissue [5,16] (LOE V, LOE II). Hepcidin interacts with the cellular Fe exporter, ferroportin, to decrease Fe flow from intestinal cells into the circulation. Hepcidin also regulates cellular Fe efflux by binding to ferroportin and inducing its internalization into cells [15]. At high concentrations, there is a reduction on Fe absorption by enterocytes and Fe recycling from senescent erythrocytes (via macrophages) [15]. In obesity, the blood levels of several proinflammatory cytokines and adipokines are increased, which mediates Fe deficiency by inducing hepcidin synthesis. As a result, obese patients have increased hepcidin expression, which may contribute to reduced Fe absorption and hypoferremia [15]. The role of hepcidin in Fe turnover remains insufficiently researched. Studies on hepcidin, especially in conditions of excess body mass, thus appear to be innovative and of high clinical importance.

In mouse studies, obesity appeared to modulate the transcription of genes involved in the regulation of Fe bioavailability. Increased hepatic hepcidin messenger ribonucleic acid (mRNA) levels and Fe accumulation at the liver and spleen have been found in obese mice [16] (LOE II). Moreover, increased leptin levels in these animals have also been observed. Leptin regulates food intake, energy expenditure, and hepcidin expression in human hepatoma cells. Thus, excessive leptin levels contribute to Fe disturbances in such mice [16]. As suggested by Skrypnik et al. in their case-control study of 212 patients with excess body mass and 145 normal-weight controls, leptin levels in obesity also correlate with levels of vascular endothelial growth factor (VEGF), which appear to impact Fe levels in the body [17] (LOE IV). However, this was the first attempt to clarify the link between VEGF and leptin serum contents, so further studies need to be performed for this conclusion to be considered reliable.

Another important marker of Fe level is ferritin. This protein reflects bodily Fe stores in healthy people, and it is an acute-phase indicator. The acute phase response (APR) is the body’s response to a disruption of homeostasis. Its occurrence is associated with the release of proinflammatory compounds [18] (LOE III). In obese patients, high ferritin serum levels are found and are ascribed to chronic, low-grade inflammatory status in these patients. Ferritin is a biomarker of iron-deficiency anemia (IDA), which is one of the most common nutritional disorders globally [19] (LOE IV).

Levels of nonheme Fe absorption are dependent on diet composition [15]. In their study of 104 randomly selected elderly respondents, Oldewage-Theron et al. suggested that obese individuals consume energy-dense and nutrient-poor diets, which leads to low Fe intake and thus increases the risk of Fe deficiency [19]. Despite the low number of participants in the study, it can be concluded that a change in diet may be the first step in improving Fe levels in obese patients. It has been also suggested that dietary Fe absorption is lower in overweight women than in normal-weight women [15].

The treatment of Fe deficiency in obese patients constitutes a significant challenge. Oral Fe supplementation is of little effect in obese children, as concluded by the review of several studies performed by Grandone et al.; however, improvements in Fe status were found in those who lost weight [5]. Supplementation combined with weight loss appears to be effective in ameliorating Fe status in obese patients [5]. Fe deficiencies connected with obesity pose huge health problems, including anemia. However, a recent cross-sectional study of 256 women of reproductive age in northern Iran suggested that the connection between excess body mass and iron deficiency anemia in women is controversial. This issue therefore needs further investigation [20] (LOE III).

On the other hand, a study by Wang et al. using *Caenorhabditis elegans* revealed that obesity may be associated with systemic Fe overload. This study provided experimental evidence supporting a previously unverified link between Fe and obesity and suggesting that Fe overload promotes the accumulation of fat; however, the underlying mechanisms are not yet fully understood [21] (LOE IV). Studies in mice have hypothesized that a high-fat diet is associated with obesity results in Fe overload [22] (LOE III). Similarly, increased Fe absorption and accumulation is associated with hepcidin, though it is not known whether this condition is due to the reduced expression of hepcidin [22].

Fe accumulation contributes to NAFLD and to nonalcoholic steatohepatitis (NASH); this was indicated by Hasebe, who performed whole-RNA sequencing on the livers of subjects in order to identify the key molecule involved in NAFLD-associated iron dysregulation [22]. Excess Fe produces hydroxyl radicals, which oxidize proteins, nucleic acids, and lipids, leading to liver fibrosis or breast cancer [22]. That study seems to indicate that not only insufficient levels of Fe, but also excess Fe, can be found in obese individuals and be harmful.

In conclusion, a range of studies have suggested that excess body mass is associated with Fe deficiency. However, there are also reports indicating that obesity may coexist with Fe excess. The relation between obesity and Fe metabolism remains unclear, and so more research is required.

### 3.2. Calcium

The connection between obesity and Ca levels has not yet been thoroughly investigated. It has been suggested that a high-fat diet does not affect Ca homeostasis [23] (LOE V). However, it does cause a low level of fast troponin isoform, which is a component of the regulatory troponin complex. The function of this troponin complex is to mediate Ca sensitivity of muscle relaxation [24,25] (LOE II). The study of Ciapaite et al. presented the exact changes in fast troponin isoforms: lower expression of the fast troponin 3 isoform, and higher expression of the fast troponin 1 isoform, was observed [24]. The study of Eshima et al. investigated the long-term and short-term effects of a high-fat diet on fast-twitch skeletal muscle; after long-term administration of the high-fat diet, a lower contractile force of this muscle was observed. This was associated with alterations in muscle fiber-type composition [25]. Ca signaling appears to be disrupted by obesity, due to the excessive production of reactive oxygen species, leading to oxidative stress [26,27] (LOE II, LOE V). In their study of human airway smooth muscle cells from obese donors, Orfanos et al. revealed these cells had increased contractibility and myosin light chain phosphorylation, which led to increased levels of intracellular Ca in them [28] (LOE III).

A study by Elaib et al. revealed that sarcoendoplasmic reticulum Ca^2+^ adenosine 5′-triphosphatase (ATPase) 3 expression was lower in obese individuals, with decreased Ca^2+^ mobilization and associated platelet hyporeactivity [6] (LOE IV). The study found a link between obesity and lower expression of human platelet calcium pump (SERCA 3). This leads to lower mobilization of Ca^2+^ in obese patients and hyporeactivity of platelets. However, Deus et al. in their study of cardiac function in obese individuals on a high-saturated fat diet did not observe significant impairments in the proteins involved in intracellular Ca^2+^ handling [29] (LOE IV).

To summarize, obesity coexists with disturbances in Ca signaling pathways. However, the association between excess body mass and body Ca levels remains underinvestigated.

### 3.3. Magnesium

Obesity is associated with disturbed body magnesium (Mg) levels, referred to as hypomagnesemia [30] (LOE III). The study of Bertinato et al. [30] compared the Mg serum levels of white and South Asian men and women living in Canada’s capital region, and examined the relationships of these levels with diabetes, glucose resistance, and BMI. The results suggested that diabetes in South Asians is positively correlated with an increased risk of Mg deficiency, and that women are more vulnerable than men when it comes to increased Mg requirements. Oxidative stress occurs in excess body mass and appears to be the cause of Mg deficiency in obesity [31] (LOE V). The review of Morais et al. [31] presents how too low or too high concentration of Mg in serum is connected to oxidative stress in obese people. Although the mechanism is not fully understood, the authors concluded that Mg is critical in metabolic reactions that cause oxidative stress. Kolisek et al. suggested that overexpression of the *PARK7/DJ-1* gene leads to oxidative stress, resulting in Mg efflux and hypomagnesemia [32] (LOE III). That study hypothesized that the disturbance in Mg homeostasis has a positive correlation with neurodegenerative, cardiovascular, and metabolic diseases. It also suggests that increased production of reactive oxygen species (ROS) is strongly associated with Mg levels. One important metabolic disturbance in obese people is adipose tissue inflammation, and Mg deficiency appears to activate proinflammatory pathways [31]. Hosseini et al. found that obese individuals have lowered levels of antioxidants and Mg, and so they may be more prone to developing CVD [33] (LOE V).

Mg is extremely important in glucose and lipid metabolism [34] (LOE III). One study [34] presents how low levels of Mg in serum lead to an increased chance of cardiovascular disease and glucose metabolic disorders in an experimental group of healthy men and nonpregnant women aged 30 to 65 years. Similarly, Mg is implicated in bronchial smooth muscle relaxation, and can be used to treat people suffering from asthma who are unresponsive to other first-line therapies [35] (LOE I). Since obese individuals often suffer from Mg deficiency, their treatment for asthma can be less effective [35].

Hypomagnesemia may underlie CVD in obese patients [30]. It has also been suggested that obesity associated with hypomagnesemia disturbs insulin functions, especially in women with excess body mass [30]. According to a Canadian study of adults from Newfoundland and Labrador, stronger associations were found between Mg intake and insulin resistance in obese individuals [36] (LOE III). This was a cross-sectional study of 2295 inhabitants of the provinces with highest rates of diabetes and obesity in Canada, conducted by Cahill et al. The study took into consideration subjects’ age, gender, caloric intake, physical activity, medication use, smoking status, menopause, and adiposity. Mg loss often occurs via osmotic diuresis in individuals with uncontrolled diabetes [34]. Hypomagnesemia in nondiabetic individuals is caused by hyperglycemia [34]. In contrast, Morais et al. hypothesized that plasma Mg levels in obese women are normal because of reduced Mg excretion with diuresis, which prevents Mg loss [37] (LOE IV). This dependency has been presented in a study of a group of 83 women [37].

Despite the fact that mineral studies have suggested that healthy men and women have similar Mg requirements, women may be at greater risk of Mg deficiency, because they usually have lower Mg intakes than men [30]. This is important in preventing hypomagnesemia during obesity. Additionally, ethnic differences also matter: the research of Bertinato et al. revealed that South Asians were more prone to Mg deficiency [30]. That is likely due to the fact that South Asians suffer from diabetes more often: their Mg intake needs are higher because of diabetes and insulin resistance [30]. Moreover, the 2015 study of Bertinato et al. also suggested that, for women only, there was an association between lower serum Mg levels and diabetes [30]. The study of Morais et al. hypothesized that Mg levels in erythrocytes from obese women were normal, probably due to Mg mobilization from other tissues—mainly osseic tissue to red blood cells, to maintain the Mg balance [31].

Skalnaya et al. presented that hair from overweight and obese people had significantly lower Mg levels, which may be associated with hypomagnesemia and obesity [38] (LOE IV, LOE II). The purpose of the study was to investigate the levels of toxic and essential trace elements in the hair of adult overweight and obese people, as well as their association with metabolic parameters. The level of minerals was evaluated and the correlation between body mass and the level of magnesium was found to be the strongest [38]. The aim of the next study was to compare the effect of endurance and endurance–strength training on mineral levels in 44 women with abdominal obesity; it was suggested that endurance–strength training decreased hair Mg levels [39].

On the basis of these studies, we can state that obesity is associated with hypomagnesemia, and that chronic subclinical inflammation seems to be an important link.

### 3.4. Trace Elements

#### 3.4.1. Copper

Cu is crucial to antioxidation mechanisms, has an important role as a coenzyme in mitochondrial homeostasis, and functions in inflammatory responses and Fe metabolism [40] (LOE IV).

Obesity is associated with an imbalance in Cu levels. Although little research has been conducted on this topic, Fan et al. found that Cu–Zn superoxide dismutase (SOD) activity was increased and total Cu circulating levels were elevated in obese children [41] (LOE III). Disturbances in Cu metabolism may trigger hypercholesterolemia by increasing ROS production, causing oxidative stress and the oxidation of low-density lipoproteins [42,43] (LOE IV). This effect of increased serum Cu intensifies the unfavorable effect of excess body mass on health and seems to be one of the many mechanisms linking obesity with oxidative stress and atherosclerosis. As indicated by Jaksic et al. in a study involving 202 children aged 7–15 years, increased Cu serum levels are positively correlated with increases in inflammatory markers, such as C-reactive protein (CRP) [44] (LOE III). This observation allows the hypothesis that increased serum Cu concentration takes part in the development of subclinical chronic inflammation in obesity. Further, Asghari et al. found in a cross-sectional analysis of 2008 candidates for bariatric surgery, aged 15–65 years, with either BMI ≥ 40 kg/m^2^ or 30 < BMI < 35 kg/m^2^ with medical comorbidities, that Cu imbalances are risk factors for cancer, CVD, and diabetes, due to these metabolic processes [45] (LOE IV). This may indicate that Cu serum levels are important in the development of increased cardiovascular risk in obesity. Equally, Calderón et al., in a study of thirty male patients with moderate to severe obesity, indicated that high Cu serum levels lead to sperm abnormalities (e.g., abnormal acrosome reactions, deoxyribonucleic acid (DNA) fragmentation, and motility) in obese men, thereby reducing sperm quality [46] (LOE IV). Increased serum Cu concentration thus seems to be a link, though only one of many, between excess body mass and reduced fertility.

Most serum Cu levels are bound to cuproproteins, such as semicarbazide-sensitive amine oxidase (SSAO) or ceruloplasmin (Cp), which are proteins that are elevated in obese patients [40]. In a review of 75 studies of the antiobesity effects of flavin-containing and copper-containing amine oxidase inhibitors, Carpéné et al. suggest that SSAO inhibitors reduce inflammatory disease, which could be useful for obesity treatment; further research, however, is required [47] (LOE V). Nevertheless, studies on SSAO inhibitors may allow new therapeutic strategies against inflammation-derived obesity complications to be developed, which makes this research direction worth developing.

Cu levels are significantly increased in the liver and adipocytes of overweight individuals. It has been suggested that serum Cu is positively correlated with serum leptin and insulin, whose level is increased in obesity [40,41,48] (LOE IV, LOE III, LOE III). Such correlations confirm that Cu takes part in the development of insulin resistance and leptin resistance—disorders crucial to obesity complications such as type 2 diabetes. Studies have suggested that this correlation is independent of age and ethnicity but is gender-dependent; both healthy and obese women have higher Cu levels than their male counterparts. This difference between females and males indicates that future therapeutic strategies against insulin and leptin resistance should be gender-specific. This indicates that obesity therapy needs to follow the individualized medicine approach.

To summarize, patients with excess body mass present elevated body Cu levels. This may be at least partially related to elevated circulating levels of cuproproteins, such as SSAO and ceruloplasmin, and higher SSAO activity in visceral fat [40].

#### 3.4.2. Selenium

Se is an essential trace element. As a key component of glutathione peroxidase and a derivative of selenoenzymes, it participates in the defenses against oxidative stress [49] (LOE V). Obesity is associated with elevated oxidative stress levels, accompanied by decreases in antioxidant enzyme activity [41].

In a cross-sectional study of 6440 men and 6849 women aged 20 years and over who participated in the US Third National Health and Nutrition Examination Survey, Zhong et al. examined the relationships between serum Se levels and anthropometric indicators, such as BMI, percentage body fat (%BF), and visceral obesity [7] (LOE III). They found that, as BMI increased, serum Se levels decreased, and that the mean differences between the extreme BMI quartiles was 4 ng/mL Se for both sexes [7]. Additionally, the %BF index was inversely correlated with Se levels in blood serum, though only in women [7]. Researchers have speculated that these outcome differences in the sexes may have resulted from differences in fat distribution between the groups [7]. This study of gender-specific differences related to Se level, alongside the studies of Cu that presented gender differences between females and males, additionally supports the hypothesis that individualized obesity diagnosis and therapy is needed depending on sex. Another study by Błażewicz et al. determined blood and urine Se levels in children aged 6–17 years and suggested that, regardless of gender, Se levels in blood and urine were lower in obese children than in normal-weight children [50] (LOE IV). This study seems to indicate that gender-specific differences in Se levels in obesity do not occur earlier than adulthood, which should be borne in mind in specialist obesity diagnostics.

We can thus conclude that excess body mass is negatively associated with blood and urine Se levels.

#### 3.4.3. Zinc

Zinc serum levels in the human body range between 70 and 120 µg/dL [51]. Plasma Zn is bound with albumin and alpha2-macroglobulin (A2M), with catalytic, structural, and regulatory functions [51] (LOE IV). Zn is an important regulator in zinc-alpha-2-glycoprotein (ZAG) homeostasis, which plays important roles in lipid metabolism and glucose homeostasis. The primary biological role of ZAG is lipid mobilization, especially in white adipose tissues [51]. As indicated by Severo et al. in a review of 58 papers on the role of Zn in ZAG metabolism in obesity, obese patients present low serum concentrations of Zn and ZAG, as well as decreased expression of the genes encoding this protein. Severo concludes that Zn acts as an important regulator of the homeostasis of ZAG and that alterations in the distribution of Zn in obese patients may affect the physiological functions of ZAG [52] (LOE V). ZAG plays an important role in lipid and glucose metabolism and seems to constitute a linkage between Zn and obesity complications.

Martins et al. performed a cross-sectional case-control study involving 80 women aged 20 to 59 years. The study presented that excess body mass correlates negatively with Zn body levels in erythrocytes as well as in plasma [51]. Begin-Heick et al. suggested in studies of male C57BL/6J-ob/ob mice and their lean controls aged 9–12 weeks that Zn levels are high in the adipose tissue, liver, and muscles [53] (LOE II). These two studies indicate that Zn level in the blood decreases in obese individuals, while Zn deposits may be found in tissues such as the muscle, liver, and adipose tissue. In the randomized, double-blind clinical trial of Payahoo et al., sixty obese patients aged 18–45 years were administered 30 mg Zn gluconate per person per day for one month, with significant decreases being recorded for body weight, BMI, and serum triglyceride levels [54] (LOE II). A similar study based on an eighteen-day supplementation of 20 mg zinc per day revealed decreased BMI, improved lipoprotein profiles, and reductions in the total cholesterol and low-density lipoprotein (LDL) fraction [54].

In summary, obesity is associated with decreased serum Zn levels. 

#### 3.4.4. Iodine

Iodine is implicated in thyroid hormone synthesis [55] (LOE V). To maintain correct iodine levels, the minimum iodide intake should be no lower than 50 µg/day [55]. Lecube et al. compared iodine levels excreted in the urine of ninety morbidly obese women, ninety women with at least eighteen months’ follow-up after bariatric surgery, and 45 healthy nonobese women. These Caucasian women were living in a free iodine deficiency area. Urine iodine concentration (UIC) was expressed as the ratio of iodine to urine creatinine (μg/g). It was found that mean UIC levels for obese women were 96.6 μg/g, whereas for the nonobese cohort, they were 173.3 μg/g. Among the obese women, those with normal levels (>100 μg/g) of excreted iodine constituted 46.6% of the entire study group. In the control group, this percentage was 83.3%. These data indicate that 54.4% of obese women had an iodine deficiency. The percentage of iodine-deficient women increased with BMI [8] (LOE IV). The study indicated that obesity is a risk factor for iodine deficiency [8], and that iodine absorption may be disturbed by the increased fat intake in obese women. Moreover, it is suspected that adipocyte cytokine secretion and insulin resistance may exert negative effects on sodium/iodide symporter (NIS) activity in enterocytes and may thus be implicated in a decrease in iodine absorption [8].

In conclusion, obese patients are at risk of iodine deficiency. Providing iodine supplementation for obese individuals at higher doses than for nonobese patients should thus be considered.

#### 3.4.5. Chromium

The biological activity of Cr is related to its valency [56] (LOE V): Its typical stable valency forms include neutral Cr (0), Cr (III) and hexavalent Cr (VI). Cr (III) is absorbed in the gastrointestinal tract and is bound by transferrin in the blood [56]. The target organs for Cr (III) are the liver and kidneys. Cr (III) is a trace element widely distributed in the human diet, and food sources include meat, nuts, cereal grains, molasses, and brewer’s yeast. It is estimated that approximately 1–2% of ingested Cr is absorbed from the diet. The inorganic form of Cr (III), chromium chloride (CrCl_3_), is eliminated more rapidly than its organic counterparts, such as niacin-bound Cr (NBC) and Cr picolinate (CrPic) [56]. Current dietary recommendations suggest a daily Cr dose of 25–45 μg for adults [57] (LOE VII).

Athletes, pregnant women, and the elderly are particularly vulnerable to Cr deficiency, due to the increased loss of Cr from intense exercise, long periods of stress during pregnancy, and the age-related inability to effectively absorb or transform inorganic Cr to its active form [58] (LOE II).

The exact effect of Cr on the body is not well understood but is believed to be associated with carbohydrate and lipid metabolism [59] (LOE II). As found in the randomized, double-blind, placebo-controlled study of Albarracin et al. on four hundred and forty-seven subjects with poorly controlled type-2 diabetes, Cr is important in promoting insulin action and controlling blood glucose [59]. Such studies on the importance of Cr in carbohydrate metabolism have increased hopes for the use of Cr in the therapy of glucose metabolism disorders, such as insulin resistance and diabetes, as complications of obesity. Since the identification of Cr (III) as the main component of glucose tolerance factor (GTF) fifty years ago, the element has been widely used for improving insulin sensitivity and weight reduction [59].

Cr deficiency is a risk factor for metabolic syndrome; patients receiving parenteral nutrition without Cr supplementation can develop diabetic symptoms, such as glucose intolerance, weight loss, and neuropathy; however, these disturbances can be reversed with Cr supplementation [60] (LOE IV).

To assess the effects of Cr (III) supplementation in combination with weight control exercises, a nine-week study was conducted on 43 young, healthy, obese women [61] (LOE II). One group was supplemented with Cr in the form of CrPic without exercise, the second group was supplemented with CrPic with exercise, the third received placebo with exercise, and the final group was supplemented with NBC. Supplements were administered orally, twice a day, in two pills containing 200 μg of CrPic, placebo, and/or NBC. The data suggested that CrPic supplementation did not reduce body weight but in fact led to weight gain. There were no changes in the placebo group, but the NBC group presented significant weight loss and reduced insulin responses to oral glucose intake. The researchers concluded that increased CrPi supplementation is contraindicated for weight loss in obese women. These results suggest that exercise training combined with NBC supplementation may be more beneficial than exercise training alone in the modification of coronary artery disease and non-insulin-dependent diabetes mellitus risk factors. According to Grant et al., these risk factors include body weight and composition, plasma glucose and insulin levels, and basal plasma lipid values (triglycerides, total cholesterol, low density lipoprotein-cholesterol, and high-density-lipoprotein cholesterol) [61]. The study of Grant et al. indicates that Cr can affect both energy expenditure during exercise and glucose metabolism, through mechanisms that have not been explored fully. Future research should thus make an attempt to clarify these mechanisms and to implement Cr supplementation in treatment for obesity and diabetes.

In the randomized, double-blinded, placebo-controlled crossover study on twenty African–American women with excess body mass engaged in a modest diet–exercise regimen, Crawford et al. found that the oral intake of 600 μg/day NBC for two months resulted in significant fat tissue loss without affecting muscle mass. Chemical blood analysis revealed no significant disorders after the study [62] (LOE II). This study confirmed the beneficial effect of Cr supplementation in the treatment of obesity. However, the study group was relatively small, which would seem to be a significant limitation of the study.

On the basis of the studies reviewed here, we can state that Cr deficiency is associated with metabolic syndrome. Cr supplementation affects body mass, but the effect of the supplementation depends on the chemical form of the Cr.

## 4. Conclusions

The studies we have reviewed suggest that excess body mass is associated with alterations in mineral levels in the body. Obesity is associated with hypomagnesemia and decreased Se and Zn levels. Cr deficiency is associated with metabolic syndrome. Obese patients seem to be at risk of iodine deficiency. On the other hand, excess body mass is associated with elevated levels of Cu.

As some studies have linked obesity with Fe deficiency while others have linked it with Fe excess, the connection between obesity and Fe level needs further investigation. Similarly, despite studies having found that obesity coexists with disturbed Ca signaling pathways, the nature of the link between excess body mass and body Ca levels still needs elucidation.

The cause–effect relation between alterations in somatic mineral levels and excess body mass remains unclear. More research is needed in this area to increase our knowledge and to come to a full conclusion.

## Abbreviations

%BF	percentage body fat
A2M	alpha2-macroglobulin
APR	acute phase response
ATPase	adenosine 5′-triphosphatase
BMI	body mass index
Ca	calcium
Cp	ceruloplasmin
Cr	chromium
CrCl_3_	chromium chloride
CRP	C-reactive protein
CrPic	Cr picolinate
Cu	copper
CVD	cardiovascular disease
DNA	deoxyribonucleic acid
Fe	iron
GTF	glucose tolerance factor
IDA	iron-deficiency anemia
LDL	low-density lipoprotein
LOE	level of evidence
Mg	magnesium
mRNA	messenger ribonucleic acid
NAFLD	nonalcoholic fatty liver disease
NASH	nonalcoholic steatohepatitis
NBC	niacin-bound Cr
NIS	sodium/iodide symporter
ROS	reactive oxygen species
Se	selenium
SOD	superoxide dismutase
SSAO	semicarbazide-sensitive amine oxidase
UIC	urine iodine concentration
VEGF	vascular endothelial growth factor
ZAG	zinc-alpha-2-glycoprotein
Zn	zinc
